# Restricted postoperative range‑of‑motion and weight‑bearing protocols prevail after lateral meniscus posterior root tear repair with anterior cruciate ligament reconstruction: A systematic review

**DOI:** 10.1002/jeo2.70546

**Published:** 2025-11-14

**Authors:** Cristiano Benelli, Edoardo Monaco, Riccardo D'Ambrosi, Alessandro Carrozzo, Gianluca Ciccarelli, Valerio Nasso, Alessandro Annibaldi, Nicola Maffulli

**Affiliations:** ^1^ Sant′Andrea Hospital, Orthopedic Unit University of Rome La Sapienza Rome Italy; ^2^ IRCCS Ospedale Galeazzi ‐ Sant′Ambrogio Milan Italy; ^3^ Dipartimento di Scienze Biomediche per la Salute Università degli Studi di Milano Milan Italy; ^4^ Dipartimento di Scienze della Vita, della Salute e delle Professioni Sanitarie Università degli Studi “Link Campus University” Rome Italy

**Keywords:** ACL reconstruction, lateral meniscus, meniscus repair, rehabilitation, root tears

## Abstract

**Purpose:**

This study systematically reviewed the literature on the postoperative rehabilitation protocols after lateral meniscus posterior root tears (LMPRTs) repair related with anterior cruciate ligament reconstruction (ACLR) regarding range of motion (ROM) exercise, weight‐bearing (WB) and brace use, to ascertain whether there are univocal views among authors.

**Methods:**

The literature was systematic searched in the PubMed (MEDLINE), Scopus, EMBASE and Cochrane Library databases. Level I–IV studies which reported data on rehabilitation protocols in patients treated surgically for combined ACLR and LMPRTs repair were included. For each study, predefined rehabilitation variables (ROM limits, WB restrictions, brace use) were extracted, and descriptive statistical analysis were conducted. The selected studies were qualitatively evaluated using the Methodological Index for Nonrandomized Studies (MINORS) score.

**Results:**

Twelve studies were included. The cohort of patients consisted of 277 participants. Among the patients, 192 were male, with a mean age at surgery of 28 ± 4 years, and a mean follow‐up of 34 ± 11 months. Regarding the initiation of ROM exercise, eight studies suggested a limited ROM (0°−90°) for 4–6 weeks, one study a limited ROM (0°−90°) for 16 weeks, while two studies reported no ROM limitations. Regarding WB, seven studies suggested non‐WB for at least 4–6 weeks, four studies suggested partial‐WB from 2 to 12 weeks, while one study suggested immediate full‐WB. Regarding the use of bracing, five studies suggested the use of bracing, one study did not recommend bracing, while in the other six, it was not specified.

**Conclusions:**

Most included studies suggested cautious rehabilitation with a restricted ROM and limited WB postoperatively. However, the protocols were heterogeneous, without consensus. Further studies are needed to clarify which protocol offers optimal outcomes after combined LPMRT repair and ACLR.

**Level of Evidence:**

Level IV.

**Abbreviations**
ACL: anterior cruciate ligament
ACLR: anterior cruciate ligament reconstruction
AOSSM: American Orthopaedic Society for Sports Medicine
IKDC: International Knee Documentation Committee
LMPRTs: lateral meniscus posterior root tears
MINORS: methodological Index for Nonrandomized Studies
PRISMA: preferred reporting items for systematic reviews and meta‐analyses
RCTs: randomised controlled trials
ROM: range of motion
SD: standard deviation
WB: weight‐bearing

## INTRODUCTION

Simultaneous meniscal lesions are common in patients with anterior cruciate ligament (ACL) injuries [22, 31, 40, 42, 53]. Lateral meniscus posterior root tears (LMPRTs), identified as radial tears within 10 mm of the posterior root attachment or posterior root avulsions of the lateral meniscus, occur in up to 14% of patients with ACL injuries [1, 4, 11, 16, 22]. LMPRTs biomechanically act as the absence of meniscus, resulting in an inability to convert axial loads into tension forces, leading to early osteoarthritis [32]. Also, LMPRTs contribute to anterolateral knee instability, resulting in a more marked pivot shift phenomenon in ACL‐deficient knee [15, 36, 50, 52]. Risk factors for associated LMPRTs and ACL rupture are a concomitant medial meniscal tear, contact sports, an increased lateral tibial slope and lateral‐to‐medial slope asymmetry [23, 43]. Different techniques have been described for LMPRTs repair, including the transtibial pull‐out technique and the all‐inside technique, eventually associated to meniscal centralisation when the root tear and coronal ligaments insufficiency leads to a meniscal extrusion [6, 10, 24].

A recent European Society of Sports Traumatology, Knee Surgery and Arthroscopy (ESSKA) Meniscus Rehabilitation Consensus provided recommendations for rehabilitation after meniscal root repairs, but there remains considerable variability in published postoperative rehabilitation strategies and management protocols [44]. The aim of the study was to systematically review the literature on postoperative management following LMPRTs repair associated to anterior cruciate ligament reconstruction (ACLR), focusing on range of motion (ROM), weight‐bearing (WB) and use of postoperative bracing. The hypotheses were that most published rehabilitation protocols would restrict knee flexion, limit the WB and frequently employ a protective knee brace.

## METHODS

The present systematic review was carried out following the preferred reporting items for systematic reviews and meta‐analyses (PRISMA) [41] guidelines and was registered with PROSPERO (registration number: CRD420251008591).

### Eligibility criteria

#### Study design

Randomised controlled trials (RCTs), case‐control studies and case series, prospective and retrospective comparative cohort studies were included. Case reports and case series that did not report data on rehabilitation protocols were excluded.

#### Participants

Patients included underwent surgery for combined ACLR and LMPRTs repair performed with a transtibial pull‑out, all‑inside suture, or shared‑tunnel technique, with or without meniscal centralisation.

#### Interventions

Studies that informed data on rehabilitation protocol in patients treated surgically for combined ACLR and LMPRTs repair.

Surgical techniques and rehabilitation protocols were collected for meniscal treatment.

#### Type of outcome measures

The outcome measures obtained from the studies were rehabilitation protocol including WB, ROM and use or not of a brace. The studies were included if reported at least one explicit postoperative rehabilitation these and provided a minimum follow‑up of 6 months.

Studies were excluded if they: combined the index procedures with additional ligament reconstructions beyond a single‐bundle ACL graft (e.g., multiligament knee reconstructions), revision ACLR surgery, osteotomies, or cartilage restoration procedures; lacked a quantitative or narrative description of postoperative rehabilitation; were case reports, technical notes, cadaveric or biomechanical studies, review articles or conference abstracts.

#### Information's sources and search

Significant literature was systematic searched by two independent reviewers (C.B. and R.D.) in the PubMed (MEDLINE), Scopus, EMBASE and Cochrane Library databases of all studies published in English to 3 March 2025, with no limitation date. A comprehensive Boolean strategy combining MeSH terms and free‐text synonyms for (1) lateral meniscus, (2) root tears, (3) meniscus repair, (4) ACL, (5) reconstruction, (6) rehabilitation was developed. Only articles published in English were included.

### Data collection and analysis

#### Study selection

Two authors (C.B. and G.C.) retrieved articles that were first screened by title and, if seemed relevant, screened further by reading the abstract. After eliminating studies not meeting the eligibility criteria, the remaining articles were evaluated for eligibility. To minimise the risk of bias, the authors reviewed and analysed all the selected articles, references, and articles excluded from the study. Any disagreement was resolved by consensus with a senior author (E.M.). Additional studies were identified by manually searching the reference lists of the included studies and relevant systematic reviews.

### Data collection process

The first two authors extracted data from the selected articles by using a computerised tool created with Microsoft Excel (Version 19.96.1, Microsoft Corp). Each article was verified again by the senior author before analysis. For each study, data were obtained regarding the patients (age, sex), their injuries (time from injury to surgery, associated injuries), the surgical technique, and employed rehabilitation protocol (WB, ROM and use of a brace).

### Level of evidence

The Oxford Levels of Evidence set by the Oxford Centre for Evidence‐Based Medicine were employed to categorise the level of evidence.

### Evaluation of the quality of the studies

The evaluation of the quality of the selected studies was conducted using the Methodological Index for Nonrandomized Studies (MINORS) score [51]. The checklist includes 12 items, of which the last four are specific to comparative studies. Each item was given a score of 0–2 points. The ideal score was set at 16 points for noncomparative studies and 24 for comparative studies.

### Statistical analysis

When the extracted quantitative parameters (age and follow‐up) were given in the articles, they were given as mean ± standard deviation (SD). In case they were not provided, alternative values such as median or range were extracted. The results of patients with and without concomitant surgeries could not be compared in a meta‐analysis given the high statistical and methodological heterogeneity in the included studies. Instead, a narrative description and comparison of the clinical results were performed.

## RESULTS

### Search results

**Figure 1:**
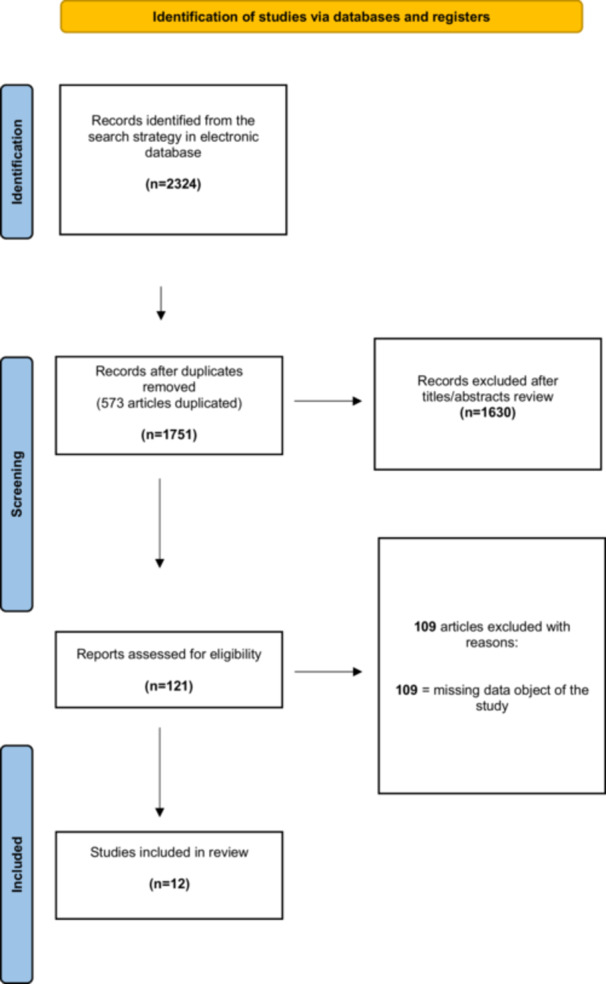
Preferred reporting items for systematic reviews and meta‐analyses (PRISMA) flow chart indicating research article inclusion for final analysis.

The electronic search produced 2324 studies. After 573 duplicates were removed, 1751 studies remained, of which 1630 were excluded after reviewing the abstracts, keeping 121 articles. According to the aforementioned inclusion and exclusion criteria, an additional 109 articles were not included. No additional studies were found by manually searching the reference lists of the selected articles. The final analysis was conducted on 12 studies [1, 26, 27, 28, 32, 46, 47, 48, 57, 58, 60, 63]. Figure 1 shows the flowchart depicting the selection process.

### Patients and study characteristics

The cohort of patients consisted of 277 participants. Among the patients, 192 were male, with a mean age at surgery of 28 ± 4 years, and a mean follow‐up was 34 ± 11 months. Table 1 shows the characteristics of the cohorts involved in the 12 selected studies and a summary of their data.

**Table 1:** General characteristics of included studies.

Reference	Year	MINORS	Level of evidence	Follow‐up—Mean ± SD (months)	M/F	Age—Mean ± SD (years)
Leafblad et al. [27]	2021	11	IV	31.2	15/6	22 ± 10
Ahn et al. [1]	2010	11	IV	18	22/3	28.8
Li et al. [28]	2022	9	IV	>24	8/7	28.0 ± 14.5
Vittone et al. [60]	2023	6	IV	7	1/1	21.5
Shekhar et al. [48]	2020	7	IV	12	3	29.7
Thomas et al. [58]	2023	13	IV	44	40/10	29.7 ± 1.0
Pan et al. [32]	2015	12	IV	37.2 ± 9.6	21/10	28 ± 10
Rocha de Faria et al. [46]	2022	5	IV	>6	3/1	33.3
LaPrade et al. [26]	2014	6	IV	24	2 (not specified)	27.5
Shekhar et al. [47]	2022	12	IV	37.4 ± 7.1	14/11	29.6 ± 6.5
Therrien et al. [57]	2023	13	IV	49.2 ± 22.8	24/26	21.4 ± 7.3
Zhou et al. [63]	2022	13	IV	>24	39/9	32.2

Abbreviation: SD, standard deviation.

The analysed studies had a mean MINORS score of 10 ± 3 (range, 5–12). Detailed results are reported in Table 1.

### Surgical techniques

Ten studies [26, 27, 28, 32, 46, 47, 48, 58, 60, 63] reported the use of a transtibial pull‐through technique, three studies [1, 57, 60] reported the use of all‐inside technique, while one study [63] performed a pull‐out in the ACLR tibial tunnel. Associated injuries and details of surgical technique are reported in Table 2.

**Table 2:** Surgery‐related details of included studies.

Reference	Injury to surgery Mean ± SD (months)	Associated injuries	Surgery technique
Leafblad et al. [27]		Radial meniscus tear	Transtibial pull‐through technique
Ahn et al. [1]	40.8	23 medial meniscus tears; 2 partial PCL injuries; 1 partial MCL tear	All‐inside technique
Li et al. [28]	2.6 ± 2.1		Transtibial pull‐through technique
Vittone et al. [60]	2.5	Ramp, PM capsular lesion	(1) Transtibial double bundle pull‐through technique (2) 2 all inside technique
Shekhar et al. [48]		MCL, posterolateral tibia osteochondral fracture	Transtibial pull‐through technique
Thomas et al. [58]			Transtibial pull‐through technique
Pan et al. [32]			Transtibial pull‐through technique
Rocha de Faria et al. [46]	12.8		Transtibial pull‐through technique
LaPrade et al. [26]		1 Bucket handle tear of the medial meniscus	Modified transtibial pull‐through technique (The sutures were secured over a button located distal and medial to the Gerdy tubercle)
Shekhar et al. [47]	7.9 ± 6.5	9 repaired medial meniscal tear (2 ramp lesion, 6 peripheral longitudinal, 1 horizontal cleavage tear)	Transtibial pull‐through technique
Therrien et al. [57]		Medial meniscal tear 38 (19%); Chondroplasty 7 (14%); MPFL repair 1 (2%); Loose body removal 1 (2%)	All‐inside technique
Zhou et al. [63]	6.1		22 = Independent transtibial pullout technique 26 = Shared ACL bone tunnel technique

Abbreviations: ACL, anterior cruciate ligament; MCL, medial collateral ligament; MPFL, medial patellar femoral ligament; PCL, posterior cruciate ligament; PM, postero medial; SD, standard deviation.

### Rehabilitation protocols

#### WB

Regarding WB seven studies [26, 27, 46, 47, 48, 60, 63] suggested nonweight bearing (NWB) for at least 4–6 weeks, then gradual return to WB; four studies [1, 28, 32, 57] suggested partial WB (PWB) from 2 to 12 weeks, while one study [58] suggested immediately full WB (FWB).

#### Range of movement

Eight studies [1, 27, 28, 32, 47, 48, 60, 63] suggested limited ROM (0°−90°) for 4–6 weeks, one study [57] indicated limited ROM (0°−90°) for 16 weeks, two studies [46, 58] reported no ROM limitations, while one study [26] did not specify ROM management.

#### Use of brace

Five studies [1, 32, 47, 60, 63] suggested the use of a brace, one study [58] did not recommend bracing, while in the six others [26, 27, 28, 46, 48, 57], bracing was not specified. Details are reported in Table 3.

**Table 3:** Postoperative management in terms of WB, ROM and Use of Brace.

Reference	WB	ROM management	Use of brace
Leafblad et al. [27]	Non‐WB for 6 weeks	0°−90° for 6 weeks, then full knee bending	
Ahn et al. [1]	Partial (50%) WB for 3 months	0°−90° for 6 weeks, then full knee bending	Limited motion brace
Li et al. [28]	Partial‐WB at 4 weeks and full‐WB at 8 weeks.	0°−90° for 4 weeks, then full knee bending	
Vittone et al. [60]	Non‐WB for 6 weeks	0°−90° for 6 weeks, then full knee bending	Brace locked at 0° for 6 weeks
Shekhar et al. [48]	Non‐WB Week 1–4, partial‐WB Week 5–6, full‐WB at Week 7	0°−90° for 4 weeks, then full knee bending	
Thomas et al. [58]	Full‐WB	Full knee bending	No
Pan et al. [32]	Partial‐WB for 2 weeks	0°−90° for 6 weeks, then full knee bending	Limited motion brace
Rocha de Faria et al. [46]	Non‐WB for 6 weeks	Full knee bending	
LaPrade et al. [26]	Non‐WB for 6 weeks; non‐WB for 8 weeks		
Shekhar et al. [47]	Non‐WB for 4 weeks, partial‐WB for another 2 weeks	0°−90° for 4 weeks, then full knee bending	Brace locked at 0° for 4 weeks
Therrien et al. [57]	Limited WB for 4 weeks	Limited loads on WB with the knee at ≥ 90° of flexion for 16 weeks	
Zhou et al. [63]	Weeks 1–3 non‐WB, partial‐ WB at 4 weeks; full‐WB at 8 weeks	Active knee flexion 0°−90° for 4 weeks, then 90°, 120° and 150° at Weeks 4, 8 and 12.	Yes

Abbreviations: ROM, range of motion; WB, weight‐bearing.

## DISCUSSION

The most important finding of this systematic review has been the marked heterogeneity and paucity of high‐quality evidence guiding postoperative rehabilitation after concomitant repair of LMPRTs and ACLR. A recent ESSKA Meniscus Rehabilitation Consensus provided recommendations for the rehabilitation after isolated meniscal root repairs [44]. Nevertheless, there remains considerable variability concerning postoperative rehabilitation strategies and management protocols.

Rehabilitation protocols must balance the risk of healing failure from early mobilisation with the risk of complications from prolonged immobilisation, such as muscle atrophy and adhesions [56]. An accelerated rehabilitation protocol could be safely used in selected patients, but it is not clear how the type of meniscal tear and the repair technique should affect the postoperative program [54].

The optimal protocol for postoperative rehabilitation after concomitant meniscus repair and ACLR is still debated [14]. Patients who underwent isolated ACLR and ACLR with meniscal repair had similar flexion and extension strength at 6 months postoperatively, suggesting that meniscal repair does not affect limb strength recovery [61]. Analogously, WB and ROM limitations after simultaneous ACLR and meniscal repair did not result in worse clinical outcomes or loss of strength at 6 months postoperatively compared to ACLR alone [7].

This systematic review summarised and focused on different aspects of rehabilitation protocols, specifically about ROM, WB and brace use.

On the one hand, ROM exercise may reduce the risk of scar tissue adhesions, arthrofibrosis and restriction of joint mobility [2, 17, 34, 37, 38]. On the other hand, early ROM exercise might damage the suture‐meniscus tissue and bone‐meniscus interface, which may lead to unfavourable meniscal healing and loss of fixation [25, 55]. Early passive ROM does not result in knee damage [18]. However, the potential negative effect of accelerated ROM exercises on the clinical outcome has not been clearly evaluated. In the early postoperative period after root repair, moderate motion is recommended to allow root fixation healing [38].

Most of the studies included in this systematic review limited WB for a few weeks after surgery. This was predictable if we consider that a meniscal root tear is a radial tear located within 1 cm of the meniscal root [10], and a radial tear repair generally requires non‐WB for the first 6 weeks [19] since those lesions experience distraction forces and increased stress with axial loading [33]. Surgeons agree that early WB may cause excessive stress and affect the healing of the meniscal repair site, whereas protective and conservative WB protocols in the early phase may be helpful to achieve more favourable meniscal healing [39]. In fact, early WB delays meniscal repairing by damaging the meniscus and placing excessive stress on a fixed root after repair, which may cause meniscal extrusion [38]. However, according to Vascellari A. et al., excessive delays in WB might have negatively impact clinical outcome because the hoop stresses associated with WB facilitate meniscal healing following repair [59]. Thus, we cannot state which type of WB protocol is superior in promoting healing, but in most studies, full‐WB was not recommended for at least 6 weeks postoperatively.

Knee bracing is commonly used postoperatively to limit knee ROM and prevent excessive varus‐valgus forces and anterior‐posterior translation and rotation of the tibia [5, 9]. According to the American Orthopaedic Society for Sports Medicine (AOSSM), the brace is used in around 85% of cases following ACLR surgery [12]. Several studies have focused on the need for braces on the knee after ACLR, with controversial results [8, 13, 35, 45, 62]. Mayr et al. compared the clinical outcomes of patients treated with and without brace after ACL reconstruction: there were no significant differences in the two groups on the International Knee Documentation Committee (IKDC) subjective and objective scores, instrumental measurement of anteroposterior laxity with KT‐1000 knee arthrometer, radiographic osteoarthritic findings and tunnel widening, in a 4‐year follow‐up [35].

A recent meta‐analysis of seven randomised clinical trials involving 440 participants found no significant differences in clinical outcomes, such as IKDC score, Lysholm score, Tegner score, visual analogic scale (VAS) pain, side‐to‐side difference and single‐leg hop test, between patients who used a knee brace and those who did not after ACL reconstruction [62]. The situation changes when meniscal repair is performed in conjunction with ACLR. Especially in the early stages of rehabilitation, knee brace protects the healing meniscal tissue by providing rotational control of the affected leg, protecting the meniscal repair from deep‐flexion weight bearing, and compensating for initial quadriceps weakness caused by pain or swelling [21, 49]. The use of braces depends on the meniscus tear pattern [54]. Kocabey et al. [20] reported excellent results using rehabilitation guidelines specific to the tear characteristics. For anterior‐posterior longitudinal tears <3 cm, they encouraged p‐WB without a brace, while for longitudinal tears >3 cm, WB in a locked brace was allowed. For complex and radial tears, they promoted brace use and p‐WB for 6–8 weeks [12]. Since root tears and radial tears are similar, an analogous principle can be used for both root tears and radial tears. However, prolonged use of a brace may delay recovery, producing excessive muscle atrophy loss of flexion ROM and increased fatigability during sports [3, 29, 30].

### Limitations

This study has several limitations. First, this systematic review was based on studies with relatively low levels of evidence. The studies included were mainly retrospective case series with a mean MINORS score of 10 ± 3 (range, 5–12), which confirmed the relatively low methodological quality of the available literature. Second, it was not possible to compare clinical outcomes, such as clinical scores, osteoarthritis evolution or meniscal healing, among different rehabilitation protocols: the studies included were retrospective case series, not comparative studies between different postoperative programs. Third, the current study comprised three different surgical techniques (10 studies [26, 27, 28, 32, 46, 47, 48, 58, 60, 63] reported the use of a transtibial pull‐through technique, three studies [1, 57, 60] reported the use of an all‐inside technique, while one study [63] reported a shared ACL bone tunnel technique). However, the main aim of this study was not to compare the clinical outcomes of different surgical techniques, but to systematically review the literature on rehabilitation protocols after surgery. Fourth, some of the patients included in the studies had other injuries in addition to LMPRTs and ACL lesion. This heterogeneity may have contributed to the difference′s outcome rehabilitation independent of protocols. Fifth, it was not possible to determine which type of rehabilitation protocol was superior.

## CONCLUSION

This present systematic review showed that there are several rehabilitation protocols after LMPRTs repair associated with ACLR regarding WB, ROM and brace use. In general, most included studies suggested cautious rehabilitation with a limited WB and a restricted ROM postoperatively. However, it was not possible to show the clear superiority of one rehabilitation protocol as the protocols considered were heterogeneous and polled analyses were not conducted.

## AUTHOR CONTRIBUTIONS

**Cristiano Benelli**: Conceptualisation; data acquisition, manuscript writing. **Edoardo Monaco**: Conceptualisation; manuscript editing. **Riccardo D'Ambrosi**: Conceptualisation; data acquisition and analysis. **Alessandro Carrozzo**: Data analysis; manuscript editing. **Gianluca Ciccarelli**: Manuscript writing. **Valerio Nasso**: Manuscript writing. **Alessandro Annibaldi**: Manuscript editing. **Nicola Maffulli**: Conceptualisation; manuscript editing.

## CONFLICT OF INTEREST STATEMENT

The authors declare no conflicts of interest.

## ETHICS STATEMENT

The authors have nothing to report.

## Data Availability

All data supporting the findings of this study are available within the paper and its Supporting Information.
